# Frank’s Sign: A Clinical Predictor of Ischaemic Strokes

**DOI:** 10.7759/cureus.81812

**Published:** 2025-04-06

**Authors:** Annie Renju, Aravinth Sivagnanaratnam

**Affiliations:** 1 Acute Medicine, Imperial College Healthcare NHS Trust, London, GBR; 2 Stroke Medicine, Northwick Park Hospital, London, GBR

**Keywords:** cardiovascular disease, diagonal earlobe crease, frank’s sign, ischaemic stroke, stroke risk factors

## Abstract

Frank’s sign is a diagonal crease on the earlobe and has been linked to cardiovascular disease. This prospective observational study aimed to assess the association between Frank’s sign and ischaemic strokes. Conducted over three months in a UK district general hospital, the study analysed data from 137 consecutive patients admitted to stroke and elderly care wards. Patient records included demographic and medical history data, and physical examinations identified the presence of Frank’s sign. Statistical analysis using the chi-squared test demonstrated a significant association between ischaemic strokes and Frank’s sign, even after excluding patients with pre-existing cardiovascular disease. These findings suggest Frank’s sign could serve as a clinical predictor for ischaemic stroke. Recognizing this sign may help healthcare providers identify at-risk individuals and implement preventative strategies for managing stroke risk factors. Further research is needed to explore additional causes and refine its predictive value.

## Introduction

Frank’s sign is a diagonal crease on the earlobe, extending from the tragus to the edge of the auricle. It was named by Dr. T. S. Frank in 1973, who noticed the presence of this sign in many of his patients with angina [1]. This physical marker has been the subject of many studies determining its potential association with various cardiovascular conditions.

The exact pathophysiology of Frank’s sign is unclear; however, various hypotheses for its cause exist. Some studies suggest that the crease is due to microvascular changes within the earlobe, aligning with underlying systemic atherosclerosis [2]. The earlobe, such as the coronary arteries, has end arteries, which lack collateral circulation. This makes them more susceptible to ischemia and changes in tissue structure. This idea reflects the observation that Frank’s sign is more prevalent in older individuals, who are also at higher risk for atherosclerotic diseases [3].

Many studies have revealed a statistically significant correlation between Frank’s sign and coronary artery disease (CAD), suggesting that the presence of this sign could be used as an easily observed indicator of cardiovascular risk [4-6]. Patients with Frank’s sign have been found to have a higher incidence of CAD. This study raises the question: could Frank’s sign also be a predictor of carotid artery disease or small vessel disease and, therefore, of ischaemic strokes?

Subtypes of ischaemic strokes (apart from the cardioembolic infarcts) are commonly caused by atherosclerosis of the carotid arteries or small vessels or perforator arteries supplying the brain, and they share similar risk factors to CAD, such as hypertension, hypercholesterolemia, and diabetes mellitus [7]. Given the established studies linking Frank’s sign to CAD, there may potentially be a similar relationship between Frank’s sign and carotid artery disease and small vessel disease, thus making it a potential clinical predictor of ischaemic strokes. Other important types of infarcts, cardioembolic infarcts, have known risk factors, such as atrial fibrillation, left ventricular thrombus, and metallic prosthetic valves.

The hypothesis of this study was that Frank’s sign is associated with an increased risk of ischaemic stroke, independent of CAD. This project aimed to determine this link by comparing the presence of Frank’s sign in patients to whether they had a history of ischaemic strokes. By investigating whether Frank’s sign could serve as a visible marker for ischaemic stroke risk, this study aimed to determine whether the physical sign could be used in predicting serious cerebrovascular events.

## Materials and methods

This study was conducted over a three-month period at Northwick Park Hospital, which is a district general hospital in London, UK. It incorporated consecutive patients admitted to the stroke and Care of the Elderly wards. The primary outcome of the study was to assess if there was a statistically significant presence of Frank's sign in patients who were admitted with or had a history of ischaemic stroke. Each patient in the study was examined for the presence of Frank's sign, which was recorded as either present or absent.

Electronic medical records were used to collect data on patients’ age, sex, and medical history, providing a thorough overview of potential risk factors and comorbidities. This information helped to understand the broader health context of the patients and to reveal any pre-existing conditions that could influence the outcomes of the study.

A potential confounder in the analysis was the presence of cardiovascular disease, which has already been linked to Frank's sign in other research. Since cardiovascular disease could interfere with assessing whether Frank’s sign is specifically linked to ischaemic stroke, after initial statistical analysis, the original dataset was restricted. Patients with a documented history of CAD were excluded, allowing for a second statistical analysis to be conducted, which aimed to isolate the association between Frank's sign and ischaemic stroke, minimising the influence of pre-existing cardiovascular conditions.

**Figure 1:**
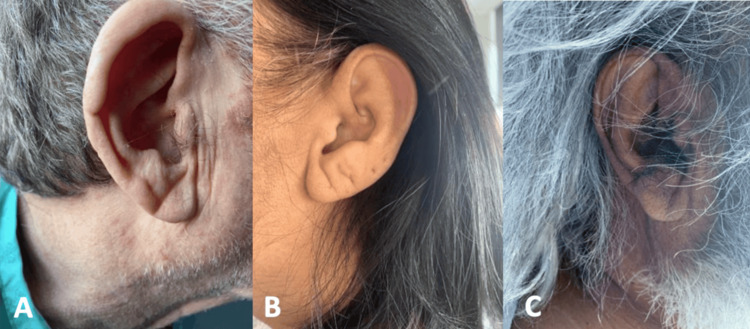
Photographs of Frank's sign in patient’s A, B, and C observed in this study

The variables of interest, including the presence of Frank's sign and a history of ischaemic stroke, were directly assessed through physical examination (Figure 1) and review of patient records. The study incorporated consecutive sampling of patients to minimise selection bias.

The study size was determined based on the patient population at Northwick Park Hospital, which includes a Hyper Acute Stroke Unit (HASU) and a Stroke Rehabilitation ward, used to identify patients admitted with ischaemic stroke. To compare this group, a similar-sized Care of the Elderly ward was selected, allowing for the inclusion of patients without a background of ischaemic stroke. The Care of the Elderly ward was chosen specifically to maintain a comparable age range between the groups, thus minimizing age-related confounding factors. The study was conducted over a three-month period due to the logistical constraints of the research project.

Patients were categorized into two groups: those admitted with or having a history of ischaemic stroke and those without any background of ischaemic stroke. These groupings allowed for a direct comparison of the presence of Frank's sign across different patient populations. The primary outcome, assessing the statistically significant presence of Frank's sign in the stroke group, was analyzed using a chi-squared test.

Missing data were addressed by utilizing multiple sources, including the hospital's local electronic records system (Cerner), General Practitioner (GP) letters, and the Summary Care Record, ensuring that each patient's medical background was comprehensively reviewed. Observations were not clustered, as each patient was only included once, even if they were readmitted during the three-month study period.

## Results

A total of 137 consecutive patients were recruited and included in the analysis, drawn from three wards at Northwick Park Hospital: the hyperacute stroke unit, the stroke rehabilitation ward, and a Care of the Elderly ward, over a three-month period. Among these, 72 patients were admitted with or had a history of ischaemic stroke. All identified patients were included in the data analysis. For all analyses in this study, a p-value of <0.05 was used to determine significance.

Baseline characteristics of all the participants, such as age and sex were recorded. To ensure the group of patients with ischaemic stroke and those without were comparable, the mean age and sex were assessed using chi-squared analysis. The average age was 77 for patients who had an ischaemic stroke, and 78 for patients who did not have an ischaemic stroke, with no statistically significant difference between groups.

The ratio of males to females were compared between the two groups of ischaemic stroke and no ischaemic stroke, showing no significant difference between the amount of males versus females in each group.

**Figure 2:**
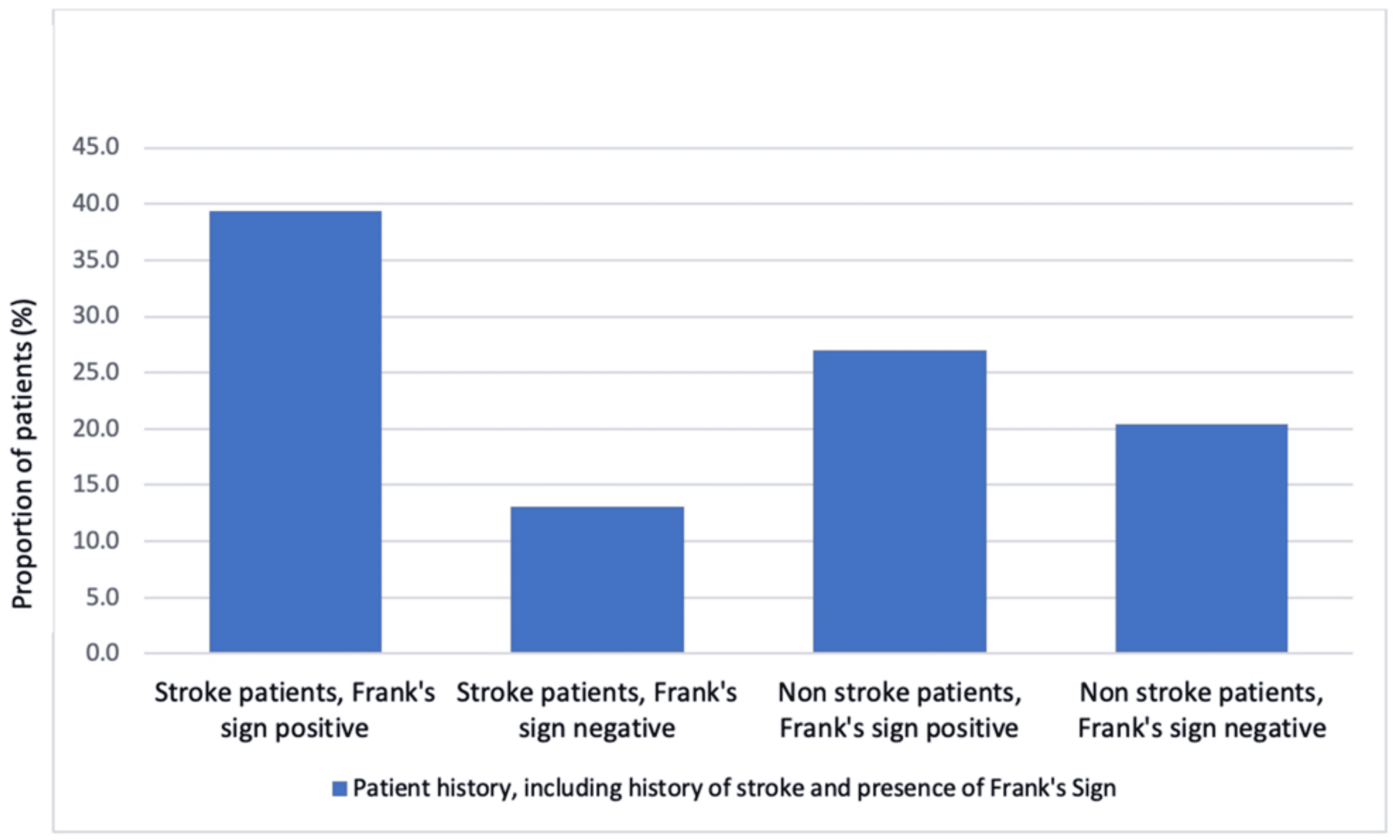
Proportion of all patients who have had a history of ischaemic stroke and/or presence of Frank's sign

Of the 72 patients who had a history of ischaemic stroke, 54 patients (75%) had Frank’s sign present (Table 1). Comparatively, out of the 65 patients in the dataset who had no history of ischaemic stroke, 37 patients had Frank’s sign (57%). The primary outcome showed a statistically significant link between ischaemic stroke and the presence of Frank’s sign (Table 2). Patients who have had an ischaemic stroke were more likely to have a positive Frank’s sign compared to those without a history of ischaemic stroke (Figure 2).

**Table 1:** Results of the analysis This table displays all patients included in the dataset, demonstrating the number of patients who had a background of ischaemic stroke and the number of patients who had a presence of Frank's sign.

Parameters	Patient’s with history of ischaemic stroke	
Patients with Frank’s sign	Present	Absent
Present	54	36
Absent	18	28

**Table 2:** Analysis of the data of patients with a history of ischaemic stroke versus without, using a p value <0.05 to determine the significance CAD: coronary artery disease

Parameters	Ischaemic stroke	No ischaemic stroke	p-value
Age	77	78	0.82
Male-Female ratio	0.87	1.21	0.45
Frank’s sign present	54	37	<0.05
Frank’s sign present, after controlling for CAD	35	25	<0.05

**Figure 3:**
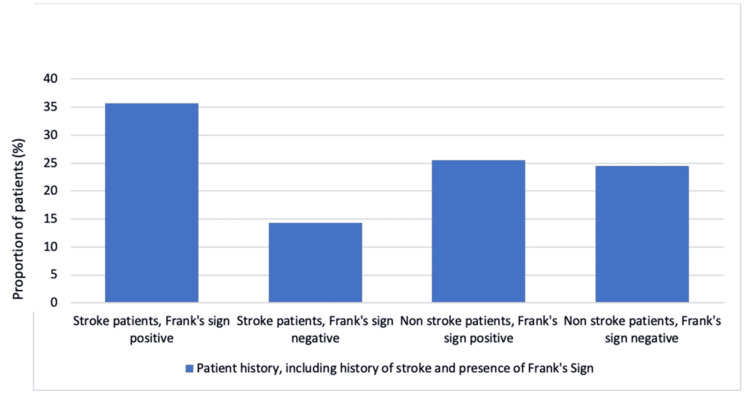
Proportion of patients, after controlling for coronary artery disease (CAD), who have had a history of ischaemic stroke and/or presence of Frank's sign

In the second analysis, patients with a history of CAD were excluded from the dataset, with 98 patients remaining (Figure 3). In this dataset, 49 patients had a history of ischaemic stroke, of which 35 patients (71%) had Frank’s sign. The other 49 patients had no history of ischaemic stroke, and among these, only 25 patients (51%) had Frank’s sign. Despite controlling for CAD, there still remained a significant association between ischaemic stroke and Frank’s sign, suggesting that the link is independent of CAD.

Among the patients with an identified Frank’s sign, data were categorized as to whether the sign was present on unilateral or bilateral earlobes. Results were then further analysed to determine if there was any association between the unilaterality or bilaterality in the ischaemic stroke versus non-ischaemic stroke groups of patients. Analysis from these data showed that there was no significant difference (p=0.80) between the two groups as to whether Frank’s sign was unilateral or bilateral.

A similar analysis was conducted to determine the significance of age and sex among patients with Frank’s sign (Table 3). In the entire dataset of 137 patients with and without ischaemic stroke, 91 patients had Frank’s sign. There did not appear to be a significant association (p=0.5) between sex and Frank’s sign.

**Table 3:** Analysis of data the of patients with Frank's sign present versus absent, using a p-value of <0.05 to determine the significance

Parameters	Frank’s sign present	Frank’s sign absent	p-value
Age	79	72	<0.05
Male-Female ratio	1.28	1.0	0.5

However, there was a significant association between age and the presence of Frank’s sign (Table 3). The mean age in patients with a positive Frank’s sign was 79, compared to 72 in patients without Frank’s sign.

## Discussion

This study highlighted a statistically significant association between the presence of Frank’s sign and ischaemic stroke, which supports the hypothesis that this physical sign can be a clinical marker of carotid artery disease or small vessel disease. The results of this study suggest that Frank’s sign could be an indicator of broader atherosclerotic risk, which just the established link to CAD, such as an indicator of ischaemic stroke. It also showed that there was a statistically significant association between Frank’s sign and increasing age. Despite the link to increasing age, a 2024 study showed that there remained a significant prevalence of Frank’s sign in young adults (age 18-25) who had a family history of chronic coronary, cerebrovascular, or peripheral vascular disease [8].

The microvascular changes leading to the development of Frank’s sign in the earlobe may reflect similar pathological processes occurring in the carotid arteries and small vessels, leading to an increased risk of stroke. These results could be relevant in clinical practice by encouraging primary and secondary healthcare practitioners to observe for Frank’s sign during routine physical examinations. In patients with a positive Frank’s sign, tighter control of ischaemic stroke risk factors, such as hypertension, diabetes, and hypercholesterolemia, may be required.

During this study, a brief literature review was conducted to see if there was any existing research determining a link between Frank’s sign and ischaemic strokes, and if so, if the results were consistent with the findings of this study. The search was carried out on PubMed, and any observational studies or case reports that focused on Frank’s sign and ischemic strokes were reviewed. One article from 2020 was found that showed a brief summary of current literature, which suggested that Frank's sign demonstrated an increase in cerebrovascular events, mainly ischaemic stroke [9]. Four articles of original research were found investigating this topic. A prospective study conducted in 1993 observed Frank’s sign in 348 patients - 116 with ischaemic stroke and 232 without [10]. It found that, in patients with ischaemic stroke, Frank’s sign was significantly related to diabetes mellitus, coronary artery disease, and non-lacunar ischaemic strokes. After controlling for CAD, there still remained a statistically significant association between Frank’s sign and ischaemic strokes.

A study from 2017 observed Frank’s sign only in patients admitted with ischaemic cerebrovascular events - ischaemic stroke and transient ischaemic attacks (TIAs) included [11]. It found that a significant number of patients who had been admitted with TIAs or ischaemic strokes had Frank’s sign present on examination.

A case study from 2020 explored malignant cerebral infarction. It noted that a patient presenting with ischaemic stroke had the presence of Frank’s sign [12]. Following thrombectomy, the patient developed massive oedema in the infarction area. The article suggested that patients with vascular disease have a higher likelihood of malignant infarction. Therefore, Frank’s sign could be used as an external marker of vascular disease and prompt close monitoring of patients with ischaemic stroke undergoing reperfusion therapy.

Finally, an observational study from 2021 found a positive association between Frank’s sign and ischaemic stroke [13]. The paper looked further into analysing the link to specific stroke subtypes. The study grouped patients with ischaemic strokes by cause of stroke - atherosclerosis, cardioembolic, and undetermined. Frank’s sign was prevalent across all subtypes.

The existing literature had conclusions aligning with the findings of this study; that is, there is a statistically significant association between Frank’s sign and ischemic stroke.

Our study has some limitations, such as the use of visual observation to identify Frank’s sign. This could be influenced by subjective judgment and affected by ear shape, piercings, or tattoos and therefore possibly affected the accuracy of identifying Frank’s sign correctly. Future studies could have multiple clinicians independently assessing each patient and comparing findings to reduce this observer bias. Another limitation was the relatively small sample size, which may prevent these results from being extrapolated to the wider population. Thirdly, although missing data were picked up on by consulting additional sources such as GP letters, the potential impact that any inaccuracies in data may have had on the study’s findings cannot be entirely ruled out. Gaps in data meant that other confounding factors, such as possible peripheral arterial disease, could not be taken into account. A further limitation was that the patients involved in the non-stroke group did not have routine CT or MRI scans at the time of data collection to rule out silent infarcts. Patients in this group were deemed to have not had a previous stroke based on clinical history and lack of findings on previous imaging. Finally, we did not analyse investigations such as carotid doppler, CT angiogram, CT head, or MRI findings to relate to Frank’s sign as it was not the primary question of this study.

The findings of this study give rise to several directions for further research. Studies with larger sample sizes and more diverse populations are needed to confirm the results of this study and could assess the impact of potential confounders, such as age, sex, and ethnicity. Additionally, more studies could assess the association between Frank’s sign specific stroke risk factors, such as cholesterol levels, blood pressure, and HbA1c, to further clarify its clinical significance. Future research could also explore whether the presence of Frank’s sign is related to the severity of carotid artery disease by utilising carotid ultrasound doppler results, or the severity of small vessel disease by using MRI brain results. This could also be an opportunity to determine if there is any significance to the depth of the diagonal earlobe crease.

There were no significant controversies caused by this study.

## Conclusions

In conclusion, the findings of this study indicate that Frank’s sign could be a useful clinical marker for identifying patients at increased risk of ischaemic stroke, aiding in early detection and prevention strategies. Observing patients for Frank’s sign during routine clinical assessments could amplify the identification of at-risk patients and prompt more targeted and rigorous management of stroke risk factors. This could lead to earlier investigations and investigations in patients with Frank's sign, such as carotid ultrasound, to detect subclinical atherosclerosis and initiate preventative measures. This may be particularly useful for patients without an obvious history of cardiovascular disease.

Furthermore, this study highlights the importance of incorporating simple, non-invasive physical examination findings into modern clinical risk stratification models. As medicine increasingly relies on advanced imaging and laboratory markers, Frank’s sign could serve as a cost-effective and quickly identifiable sign that warrants further evaluation. Future studies with more diverse populations are needed to validate the results of this study and to determine the true predictive value of Frank's sign, as well as to evaluate whether the severity or depth of the diagonal earlobe crease correlates with the degree of atherosclerosis or stroke severity.
